# The patient perspective on remote monitoring of implantable cardiac devices

**DOI:** 10.3389/fcvm.2023.1123848

**Published:** 2023-03-02

**Authors:** Henrike A. K. Hillmann, Claudius Hansen, Oliver Przibille, David Duncker

**Affiliations:** ^1^Hannover Heart Rhythm Center, Department of Cardiology and Angiology, Hannover Medical School, Hannover, Germany; ^2^Heart and Vascular Center, Hospital Neu-Bethlehem, Göttingen, Germany; ^3^Cardioangiologisches Centrum Bethanien (CCB), Device Clinic, Frankfurt, Germany

**Keywords:** remote monitoring, cardiac implantable electronic devices, pacemaker, implantable cardioverter defibrillator, sudden cardiac death

## Abstract

**Aims:**

Remote monitoring for patients with cardiac implantable electronic devices (CIEDs) is well established in clinical routine and recommended by current guidelines. Nevertheless, data regarding patients’ perceptions are limited. Therefore, this study aims to analyze the patient perspectives on the remote monitoring of cardiac devices in Germany.

**Methods and results:**

Patients with CIEDs and remote monitoring of all current manufacturers from three German centers were asked to participate. The questionnaire consisted of 37 questions regarding the patients’ individual use and perspectives on remote monitoring. Survey participation was anonymous and on a voluntary basis. A total of 617 patients (71.6% men) participated. Most patients reported feeling well informed (69.3%) and reported having unchanged or improved coping (98.8%) since the start of remote monitoring. At least 39.7% of patients experienced technical problems regarding the transmitter, whereas most patients (60.3%) reported that they never noted technical issues. Older patients had significantly less interest than younger patients in using their own smartphones for data transfer (*p* < 0.001).

**Conclusion:**

Patients with remote follow-up of CIED reported that they felt well informed about the remote monitoring approach. Remote monitoring can support coping with their disease. With remote monitoring, patients experienced a prolongation of intervals of in-person follow-up visits, and especially younger patients would appreciate smartphone-based data transfer of their CIEDs.

## 1. Introduction

Remote monitoring in patients with implantable cardiac devices is associated with reduced hospitalization rates (1–3), increased survival rates (2, 3), and a reduction of necessary healthcare resources (1, 4). It is non-inferior to in-person follow-up visits regarding patients with single or dual chamber pacemaker (PM) devices and activated automatic threshold algorithms (5), as well as patients with an implantable cardioverter-defibrillator (ICD) (4, 6–8). Thus, remote monitoring is recommended for patients with a cardiac implantable electronic device (CIED) having problems attending in-person follow-up visits or struggling with chronic device-related problems to expand the time between two in-person follow-up visits (9, 10). Patients with an ICD managed *via* remote monitoring experience fewer inappropriate ICD shocks (8, 11, 12), which is an important aspect as ICD shocks are known to increase patient mortality (13). Therefore, current guidelines recommend remote monitoring for ICDs to minimize the occurrence of inappropriate shocks (14). Moreover, remote monitoring can reduce the time between an event and reaction (11, 15, 16), with a concomitant decrease in adverse outcomes (3, 17) due to clinical problems or technical issues. Furthermore, remote monitoring is cost-effective (18, 19), reduces workload (20), and is time-effective (21). Most patients have an unchanged or improved quality of life after device implantation (22) and are satisfied while using remote monitoring (23). Nevertheless, detailed information regarding patients’ perceptions and individual concerns toward remote monitoring as well as possible future perspectives is limited. This study aims to analyze the patients’ perception of remote monitoring and possible future perspectives on remote monitoring of CIEDs.

## 2. Methods

Patients with remote monitoring of a CIED of all current manufacturers in three German centers (Hannover Heart Rhythm Center, Hannover; Heart & Vascular Center, Göttingen; CCB, Frankfurt) were invited to fill in a patient questionnaire sent by mail. Survey participation was anonymous and on a voluntary basis. Questionnaires that had been returned between July and November 2021 were included in the analysis. The present study was conducted in compliance with the Declaration of Helsinki.

### 2.1. Questionnaire

The questionnaire consisted of 37 questions regarding patients’ baseline characteristics, individual use, and opinion on remote monitoring, as well as the patients’ opinion regarding future remote monitoring perspectives (refer to Appendix Table S1). Remote monitoring includes three concepts (9): (1) remote follow-up with scheduled interrogations of the CIED, (2) remote monitoring with unscheduled data transfers due to upcoming events or alerts, and (3) patient-initiated follow-ups with unscheduled data transfers due to symptomatic arrhythmias or other issues. Hereafter, the term “remote monitoring” is going to include all those three aspects. Due to the type of question, there were three response options: single-choice, multiple-choice, or free text. The questionnaire could be filled in paper-based or web-based, according to patients’ preferences. No questions were set as mandatory. Patients with a cardiac resynchronization therapy defibrillator (CRT-D) were included in the category of patients with ICD, whereas patients with a cardiac resynchronization therapy pacemaker (CRT-P) were included in the group of patients with PM.

### 2.2. Statistical analysis

Categorial data are presented as numbers and percentages. Percentages given were calculated due to the total amount of given answers per question (refer to Appendix Table S1). Continuous data are presented as median (P25;P75). Wilcoxon test, Mann–Whitney U-test, or Kruskal–Wallis test was used for between-group comparisons, as appropriate. Statistical data analysis was performed using SPSS (version 27, IBM, Armonk, United States). *P*-values of < 0.05 were considered statistically significant.

## 3. Results

A total of 617 patients (437 men, 71.6%) from three electrophysiological centers in Germany participated between July and November 2021. Two hundred and twenty-five questionnaires were received paper-based, and 392 questionnaires were received web-based.

### 3.1. Baseline characteristics

Baseline characteristics are presented in Table 1.

**Table 1:** Baseline characteristics of the patients.

Parameter	*n* = 617 (%)
Male, *n* (%)	437 (71.6)
Age range, *n* (%)	
10–29 years	7 (1.1)
30–49 years	50 (8.1)
50–69 years	262 (42.8)
70–89 years	279 (45.6)
90–99 years	14 (2.3)
Device, *n* (%)	
ICD incl. CRT-D	424 (70.3)
PM incl. CRT-P	72 (11.9)
Implantable loop recorder	107 (17.7)
Residence, *n* (%)	
City	337 (55.8)
Rural area	252 (41.7)
Other	15 (2.5)

CIED, cardiac implantable electronic devices; ICD, implantable cardioverter-defibrillator; CRT-D, cardiac resynchronization therapy defibrillator; CRT-P, cardiac resynchronization therapy pacemaker; PM, pacemaker.

### 3.2. Remote monitoring and transmitter handling

**Figure 1:**
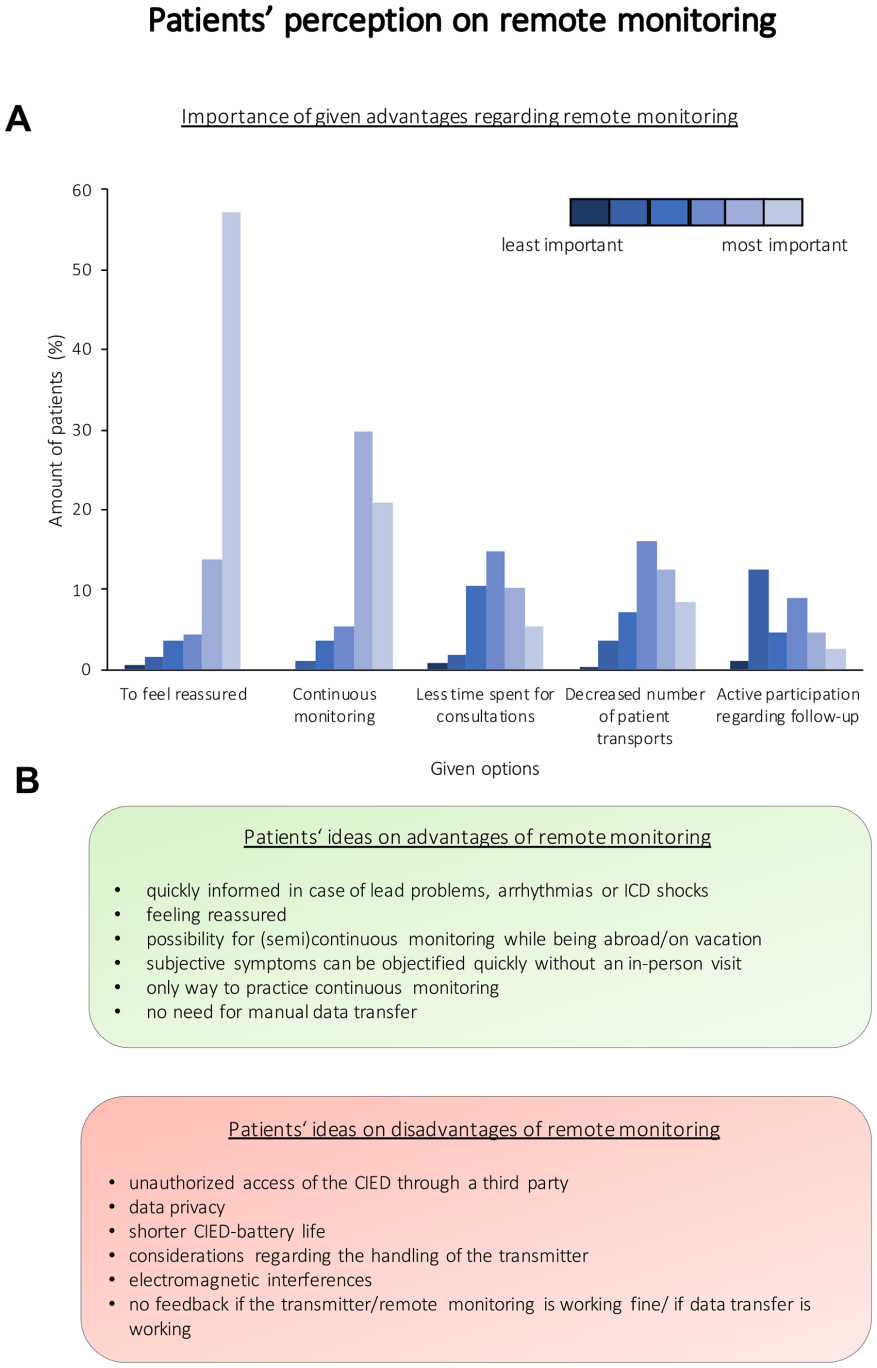
Patients’ perception of remote monitoring. **(A)** Importance of given advantages regarding remote monitoring. As patients had the possibility to choose multiple answers, they had to sort the given answers according to their importance. The importance is represented in colors. **(B)** Patients’ ideas on the advantages and disadvantages of remote monitoring. ICD = implantable cardioverter-defibrillator; CIED = cardiac implantable device.

**Figure 2:**
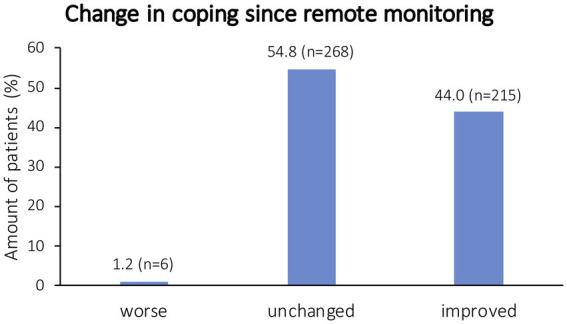
Patients’ answers on the change regarding coping since the use of remote monitoring.

Most patients said that they felt well informed about this tool (fully agree: 27.2%, *n* = 101; agree: 42.1%, *n* = 156; neutral: 26.4%, *n* = 98; do not agree: 4.6%, *n* = 17; do not agree absolutely: 1.1%, *n* = 4). The majority of patients answered not to have any concerns in regard to their telecardiological monitoring (*n* = 436, 92.0%), while 38 patients indicated having concerns regarding this aspect (8.0%). Patients’ view on remote monitoring is summarized in Figure 1. To receive information about remote monitoring and the transmitter (multiple answers possible), all patients would prefer a personal conversation. One hundred and sixty-two patients (43.9%) would rather have a brochure, whereas 136 patients (36.9%) would prefer an instruction manual. Mobile applications, instruction videos, websites, or a set of frequently asked questions were chosen by 87 (23.6%), 85 (23.0%), 79 (21.4%), and 75 (20.3%) patients, respectively. Since using remote monitoring, most patients answered to experience an improved or unchanged coping with their disease (Figure 2). Asked for individual reasons, patients who answered to have improved coping since using remote monitoring stated to feel better monitored, reassured, and/or secure. Patients with a worse coping, e.g., stated to experience a higher focus on the disease than before and that everything feels too complicated. Patients reported that remote monitoring resulted in a prolongation of intervals of in-person follow-up visits for device interrogation as well as in-person follow-up visits at the attending cardiologist (Table 2). Technical problems regarding transmitter handling were reported by 196 patients (39.7%), whereas 298 patients (60.3%) never noted any technical problems. In case of questions regarding the transmitter, most patients refer to their cardiologist (*n* = 286; 62.3%) or another medical specialist (*n* = 75; 16.3%). Other chosen contacts were the manufacturer (*n* = 42; 9.2%), relatives (*n* = 15; 3.3%), emergency service (*n* = 5; 1.1%), an association (*n* = 1; 0.2%), or nobody (*n* = 35; 7.6%). Four hundred and twenty-eight (84.9%) of the participants indicate to handle their transmitter on their own without any assistance from a third person, whereas 76 participants (15.1%) answered to get assistance. If assistance was given (multiple answers were possible), most patients chose “a member of the family” as a helping person (*n* = 66; 86.8%). Most participants never take the transmitter with them if they go on a trip for more than 24 h (*n* = 285, 65.1%). Other chosen answers were less often (“Yes, always,” *n* = 71, 16.2%; “Yes, in cases of >48 h,” *n* = 19, 4.3%; “Yes, in case of >1 week,” *n* = 45, 10.3%; “Other,” *n* = 18, 4.1%). The majority answered not to consider the transmitter a limitation (*n* = 445, 95.3%), whereas 22 patients answered to see the transmitter as a limitation (4.7%). Individual reasons given were cumbersome equipment, permanent illumination in the bedroom, having to take their transmitter with them when leaving home, or the necessity of an internet connection.

**Table 2:** Follow-up intervals for CIED interrogation or cardiologist consultation before and since the start of remote monitoring.

Follow-up intervals	Before remote monitoring	Since remote monitoring	*p*-Value
Interval of CIED interrogations	6.0 (4.0; 6.0) months	6.0 (6.0; 12.0) months	<0.001
Interval of cardiologist consultations	6.0 (6.0; 10.0) months	6.0 (6.0; 12.0) months	<0.001

CIED, cardiac implantable electronic devices.

### 3.3. Future perspectives

**Figure 3:**
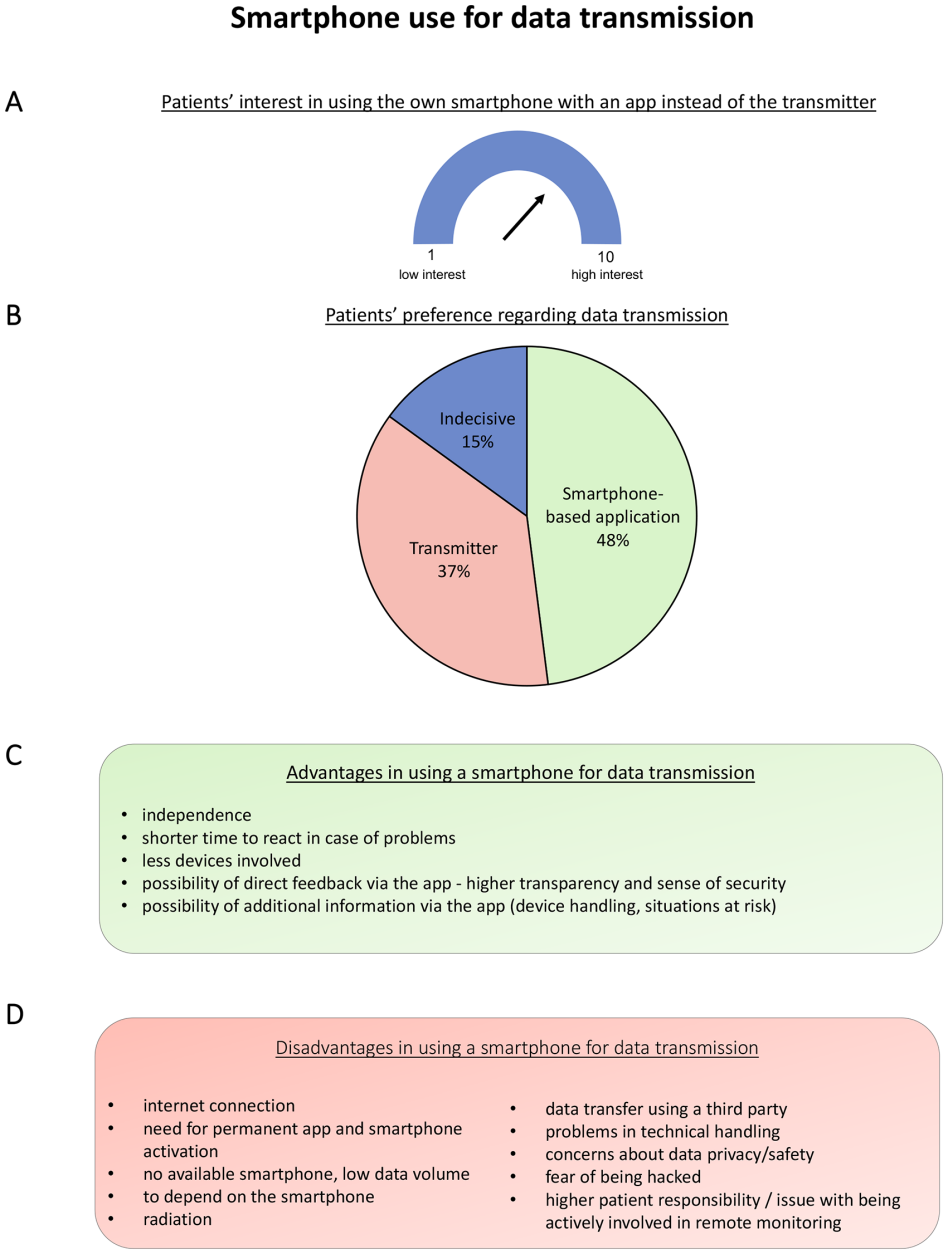
Patients’ opinions regarding the use of a smartphone for remote monitoring. Patients’ interest in using their own smartphone with an application instead of the transmitter for data transmission. Patients had to choose between 1 and 10 with 1 meaning “no interest” to 10 meaning “high interest”–median (P25; P75) 7.0 (2.0; 10.0) **(A)**. Patients’ responses to the question if they would, asked today, prefer a smartphone with an application or a transmitter next to their bed for data transmission **(B)**. Advantages **(C)** and disadvantages **(D)** of using a smartphone for remote monitoring.

A total of 383 patients answered to have a smartphone (73.0%), whereas 142 do not have a smartphone (27.0%). Most patients answered to use their smartphone for phone calls (*n* = 373, 96.9%), taking pictures (*n* = 305, 79.2%), or mobile internet use (*n* = 315, 81.8%; multiple answers possible). Other answers were less frequent (to play games: *n* = 73, 19.0%; other: *n* = 68, 17.7%). Two hundred and thirty-one patients reported using their smartphone several times a day (60.2%), whereas 86 patients reported permanent use (22.4%), 29 patients used several times a week (7.6%), 24 patients used one time a day (6.3%), and 14 patients (almost) never used their smartphone (3.7%). Two hundred and ninety-nine (81.5%) patients answered to download applications. Patients’ answers on the possible use of a smartphone for data transfer regarding remote monitoring are summarized in Figure 3.

**Figure 4:**
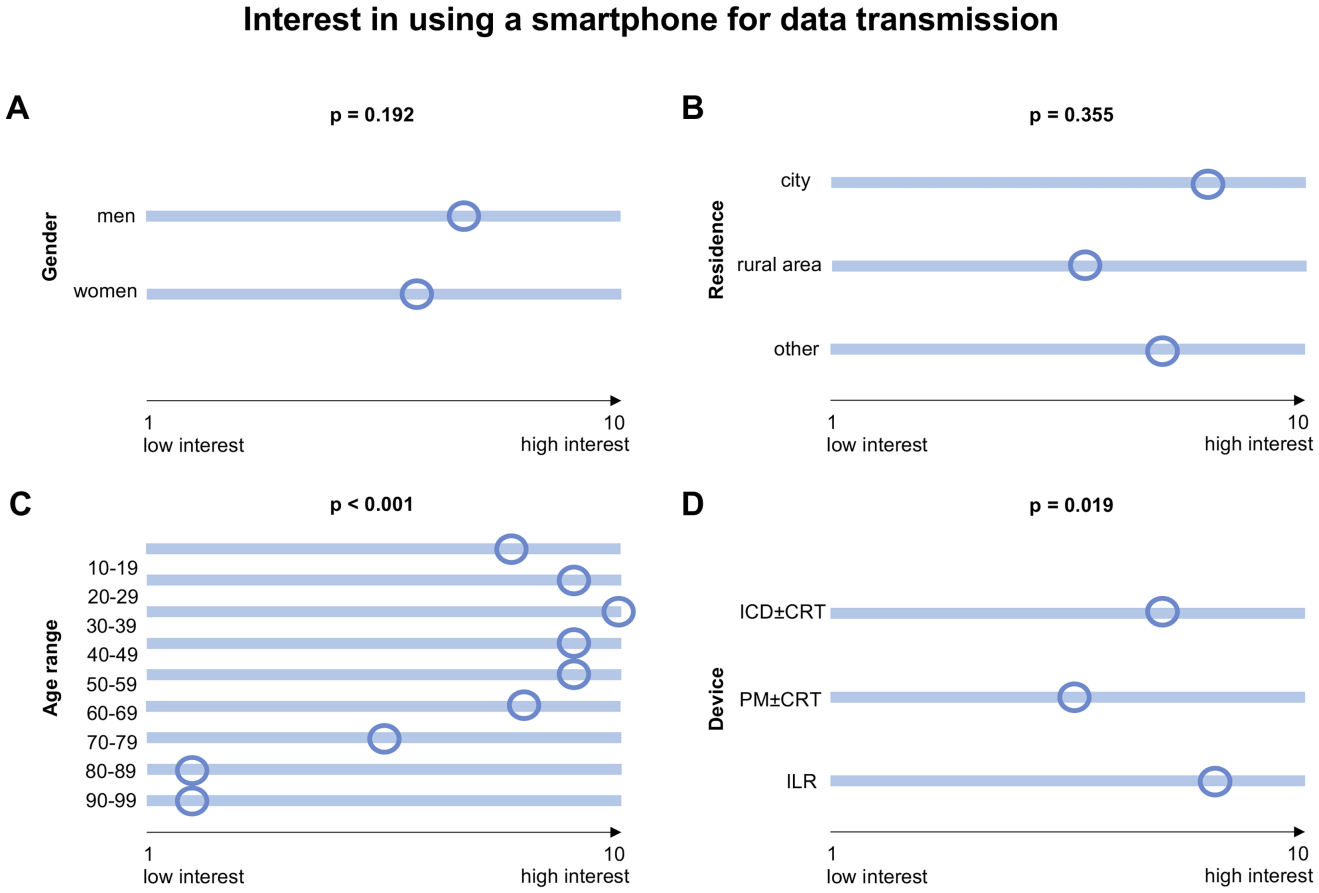
Interest in using the own smartphone instead of the transmitter for data transmission, divided into groups regarding different parameters **(A–D)**. Patients had to choose between 1 and 10 with 1 meaning “no interest” to 10 meaning “high interest.” *p*-values of < 0.05 were considered statistically significant. The small circles represent the median. ICD = implantable cardioverter defibrillator; PM = pacemaker; CRT = cardiac resynchronization therapy; ILR = implantable loop recorder.

Younger patients had a significantly higher preference for using smartphone-based remote monitoring instead of a transmitter (*p* < 0.001). There was no difference when analyzing gender (*p* = 0.192) or residence (*p* = 0.355). Details on the interest in using the own smartphone instead of the transmitter are shown in Figure 4.

Most patients stated to have concerns regarding data safety (*n* = 156, 40.8%). Other concerns were battery consumption of the implanted device (*n* = 109, 28.5%) and the smartphone (*n* = 57, 14.9%), memory capacity (*n* = 79, 20.7%), data consumption (*n* = 72, 18.9%), and other concerns (*n* = 37, 9.7%). Other concerns were fear of mobile phone obsession, the necessity to always have their phone with them and always be online, fear of being hacked (data, mail, and implanted device), and the fear of complex handling for older patients. One hundred and forty-five patients (38.0%) answered to have no concerns.

**Figure 5:**
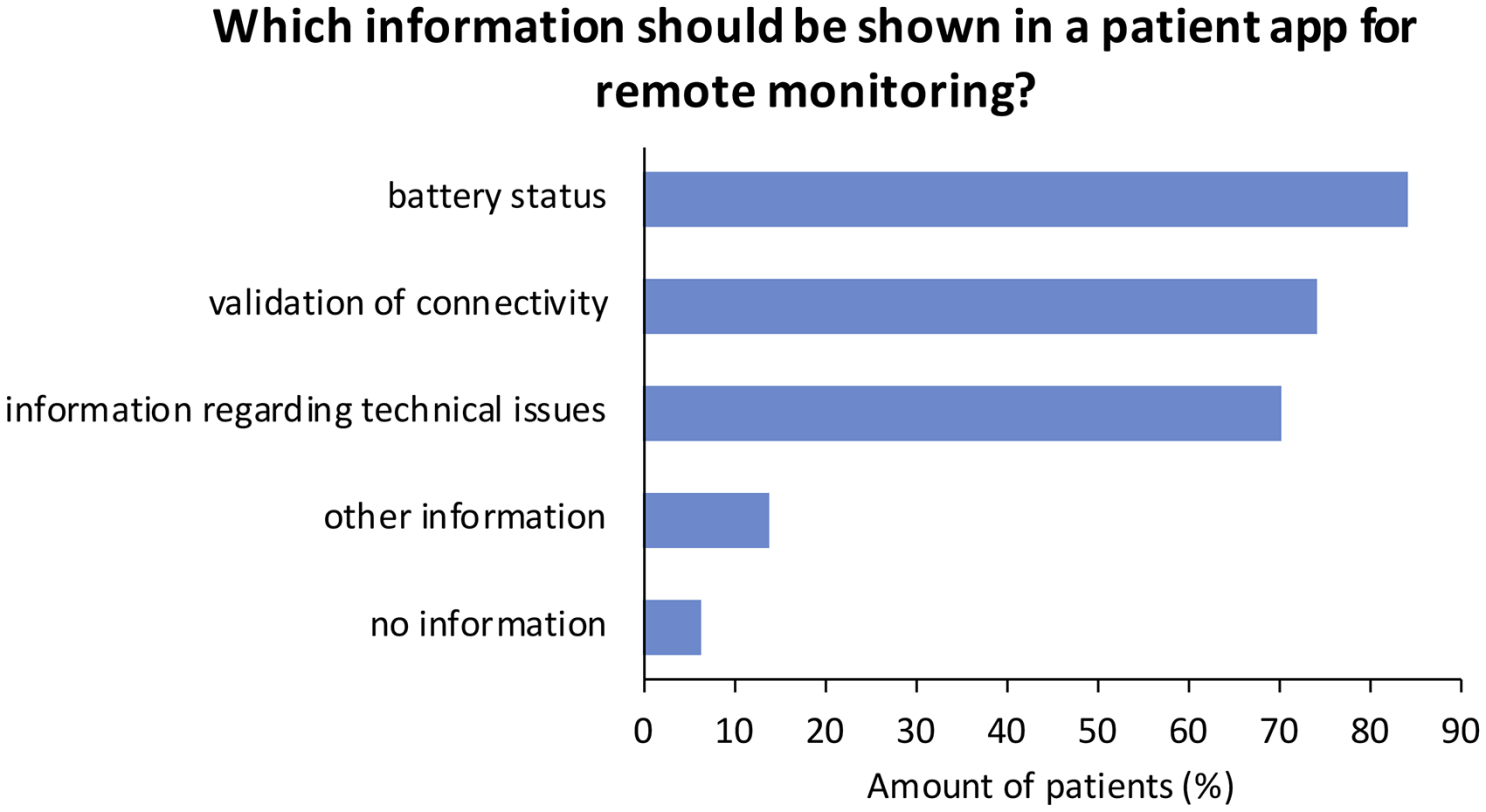
Patient’s responses on information that should be shown in an application regarding remote monitoring of CIEDs. Multiple answers were possible. CIED = cardiac implantable electronic device.

Asked for information that should be shown in a smartphone-based application (multiple answers possible), most patients chose “battery status” of the implantable device (*n* = 289, 84.3%), “validation of connectivity” (*n* = 255, 74.3%), or “information regarding technical issues” (*n* = 241, 70.3%; Figure 5). Twenty-two patients (6.4%) answered “no information,” whereas 47 patients (13.7%) answered to wish for “other” information.

## 4. Discussion

This is the first survey regarding the patient perspective on remote monitoring in Germany, not only focusing on patient satisfaction but also addressing advantages, concerns, and future perspectives perceived by the individual patient.

The key findings of this analysis are as follows:

- Most patients fully agree (27.2%) or agree (42.1%) to feel well informed about remote monitoring and answered to have an unchanged (54.8%) or improved (44.0%) coping since the start of remote monitoring.
- The majority of patients (60.3%) never experienced any technical issues related to the remote monitoring system, whereas 39.7% of the patients reported technical issues in the course of remote monitoring.
- Patients experienced a significant prolongation of intervals of in-person follow-up visits since the use of remote monitoring.
- Approximately, 48.3% of the participants would appreciate smartphone-based data transfer of their CIED.
- Nevertheless, most patients (62.0%) affirmed to have concerns about smartphone-based data transmission [addressing, e.g., data safety (40.8%), battery consumption of the implanted device (28.5%) and smartphone (14.9%), the memory capacity (20.7%), and data consumption (18.9%)].

### 4.1. Remote monitoring and transmitter handling

In this survey, previous data regarding the reduction of in-person follow-up visits due to remote monitoring (5, 20) could be confirmed due to a significant reduction (*p* < 0.001) of in-person follow-up visits not only regarding interrogations of the CIEDs but also regarding the number of in-person visits of the attending cardiologist. In accordance, patients in this survey chose less time spent on consultations as an important aspect regarding remote monitoring. Nevertheless, reassurance and continuous monitoring with the possibility of a fast objectification of symptoms and the possibility of being quickly informed in case of problems were named as the most important advantages due to remote monitoring. As patient involvement in coping becomes more important (24) and patient-reported outcome instruments to quantify the quality of life are evolving (25), this is an important point to consider. Accordingly, most patients answered to experience unchanged (54.8%) or improved (44.0%) coping since the start of remote monitoring.

Due to the COVID-19 pandemic with concomitant restrictions regarding the possibility of in-person visits and face-to-face follow-ups, remote monitoring of CIEDs became more important, and a significant increase in the number of patients using remote monitoring for their CIEDs has been observed (26). The REMOTE-CIED randomized trial reported no differences in “patient-reported health status and ICD acceptance” between patients with remote monitoring follow-ups and in-clinic follow-ups (7). Recent studies have evaluated the quality of life in patients with ICDs and results have not shown a significant decrease in quality of life, in general (22, 27, 28). Nevertheless, it has been shown that inappropriate shocks in patients with ICDs reduce the quality of life (22). Remote monitoring, therefore, has the potential to additionally increase the quality of life and reassurance in patients with a CIED and especially ICDs.

Most patients are satisfied using remote monitoring and prefer remote monitoring over in-person follow-ups (29–31). Concerns, disadvantages, and reasons for a worse coping regarding remote monitoring recorded during this survey such as data concerns, the fear of unauthorized access regarding the CIED through a third party, as well as the insecurity about the transmitter functionality may help physicians and manufacturers to further improve and evolve remote monitoring. On the one hand, focusing on patient education regarding remote monitoring and the handling of the transmitter may reduce individual anxiety and the number of patients unsatisfied with remote monitoring and, therefore, improve the quality of life. On the other hand, the idea to manage follow-ups *via* remote monitoring should be discussed with eligible patients individually due to shared decision-making.

Concerning patient education, patients answered to prefer a brochure (43.9%), or instruction manual (36.9%) as the medium to get information about remote monitoring and the transmitter but also accepted other tools such as mobile apps or websites with 23.6 and 21.4%, respectively. Brochures and instruction manuals should already be available for every patient. The development of other tools such as apps or patient websites could further improve the patients’ educational level (32, 33).

The majority of patients in this survey did not consider the transmitter as a limitation (95.3%) and 60.3% of the patients answered that they never experienced any technical issues. Nevertheless, 39.7% of the patients stated to note technical issues. This should be taken seriously, as a working transmitter is crucial for continuous data transfer.

Most patients in this survey answered to refer to their attending cardiologist or other medical specialists in case of questions. As the ones most referred to, cardiologists and cardiology nurses should be carefully trained regarding troubleshooting and use of the different transmitters. Physicians responsible for interrogations should be aware of patients who do not send data and should subliminally reach out to those patients. Doing this could not only improve the patient’s quality of life but also further improve the continuity and quality of remote monitoring while minimizing adverse events due to insufficient monitoring.

### 4.2. Future perspectives

The majority of patients (73.0%) answered to use a smartphone, while only a minority of patients were younger than 60 years (*n* = 159). Thus, age is not necessarily a restriction regarding the use of smartphone-based techniques (34). Accordingly, most patients participated in this survey *via* an online platform and not *via* the paper-based form. A total of 48.3% of patients answered to prefer a smartphone-based application to a transmitter next to their bed for data transfer. Previous studies have shown that smartphone-based remote monitoring has the potential to improve the success rates of scheduled data transfers (35) and to improve patients’ compliance and connectivity (36). Therefore, application-based data transfer may improve the management of remote monitoring in several ways: Feedback regarding successful or unsuccessful data transfers as well as a schedule for measurements regarding remote follow-ups could be integrated. Patients could be directly informed in case of events and possible next steps could be recommended within such an application. Moreover, patient education could be further enhanced with information regarding remote monitoring or specific situations. According to the patients who participated in this survey, information on battery status, validation of connectivity, and information regarding technical issues should be taken into consideration when developing such a tool. Nevertheless, patients’ concerns and disadvantages regarding such an idea should not be ignored. Especially, data safety is an important concern that should be noted (37). Not all patients will have access to data transfer and remote monitoring *via* a smartphone. As shown in this survey, this may especially be relevant for older patients. Since current guidelines recommend remote monitoring for patients with difficulties attending in-person visits, this might be an issue that should be targeted.

As remote monitoring is known to reduce hospitalizations due to heart failure, application-based alerts can help patients and physicians to adapt and improve heart failure management (3, 4, 6, 7, 38, 39). CIEDs may be helpful to contribute to optimized heart failure management. Nevertheless, studies have shown that with one parameter alone there may be no significant improvement in outcomes (38, 39). Algorithms including multiple parameters may be more appropriate but have to be evaluated (40–42).

## 5. Limitations

This survey has several limitations regarding the respondents as well as the designed questionnaire. As the survey was anonymous and on a voluntary basis, the study population includes an expectable selection bias and baseline, as well as follow-up data on the patient group are scarce. Having only included patients already receiving remote monitoring, the study design did not provide a control group and does not allow comparisons of patients with remote monitoring vs. those without remote monitoring care, and thus, recall bias may be present. Nevertheless, the overall sample size provides important insights into a large cohort of patients followed-up by remote monitoring.

## 6. Conclusion

In this national patient survey, remote monitoring led to a prolongation of intervals of in-person follow-up visits, while most patients reported unchanged or improved coping since the start of remote monitoring and answered to be open to new technologies regarding data transfer. Nevertheless, some patients reported concerns about remote monitoring as well as worse coping since the start of remote monitoring. The medical specialist was named as the person mostly referred to in case of problems. Thus, both the patients’ and the physicians’ education are important to improve the continuity and quality of remote monitoring while minimizing adverse events due to insufficient monitoring and therefore to improve the quality of life in patients with CIEDs and remote monitoring.

## Data availability statement

The raw data supporting the conclusions of this article will be made available by the authors, without undue reservation.

## Ethics statement

Ethical review and approval was not required for the study on human participants in accordance with the local legislation and institutional requirements. All participants volunteered to participate in this anonymous data collection.

## Author contributions

HH acquisitioned the data, performed the analysis, interpreted the data, and drafted the manuscript. CH and OP revised the manuscript and provided substantial intellectual content. DD analyzed the data and drafted and revised the manuscript. All authors contributed to the article and approved the submitted version.

## Conflict of interest

Abbott Medical supported the conduction of the survey by executing the postage of the questionnaires to patients and designing the online questionnaire, and also provided open access funding, but did not have any influence on patient selection, data analysis, nor writing or editing the manuscript. OP received lecture honorary from Abbott Medical, Biotronik, Medtronic, Zoll. DD received modest lecture honorary, travel grants, and/or a fellowship grant from Abbott, Astra Zeneca, Biotronik, Boehringer Ingelheim, Boston Scientific, Bristol Myers Squibb, CVRx, Medtronic, Microport, Pfizer, and Zoll.

## Publisher’s note

All claims expressed in this article are solely those of the authors and do not necessarily represent those of their affiliated organizations, or those of the publisher, the editors and the reviewers. Any product that may be evaluated in this article, or claim that may be made by its manufacturer, is not guaranteed or endorsed by the publisher.
