# Supplementary material for: The patient perspective on remote monitoring of implantable cardiac devices
Source: Front Cardiovasc Med. 2023 Mar 2;10:1123848. doi: 10.3389/fcvm.2023.1123848 (PMC10017432; doi:10.3389/fcvm.2023.1123848)
Supplement: Supplementary file 1 [file Table_1.DOCX]

| **Question Type (QT); Response Rate (n), n=617** | **English version** | **Answers to choose** |
| --- | --- | --- |
| Q1 (SC; 612) | Have you ever heard about remote monitoring before participating in this survey? | yes/no |
| Q2 (SC; 610) | Please state your gender | female/male |
| Q3 (SC; 612) | How old are you or is the patient you are speaking for? | 0-9/10-19/20-29/30-39/40-49/50-59/60-69/70-79/80-89/90-99/≥100 |
| Q4 (SC/text; 604) | Where do you live? | city/countryside/others (text) |
| Q5 (SC; 603) | Which type of CIED have you been implanted with? | defibrillator/pacemaker/heart monitor |
| Q6 (SC; 610) | Do you participate in telecardiological monitoring? | yes/no/don’t know |
| Q7 (MC; 371) | Do you feel well informed about telecardiological monitoring? | fully agree/agree/neutral/don’t agree/fully don’t agree |
| Q8 (MC/text; 369) | What kind of medium what you prefer to receive information regarding remote monitoring and remote monitoring equipment (e.g. transmitter)? | brochure/instruction manual/set of frequently asked questions/website/mobile app/instruction video/others (text) |
| Q9 (MC, sort in order of importance; 366) | What kind of advantages would you ascribe to remote monitoring (if you would like to choose multiple answers please sort regarding the importance, 1 = the most important) | to feel calm/continuity of monitoring/fewer trips to the hospital/less time spent for consultations or in-patient visits/patients’ participation regarding the follow-ups/no additional benefit / others |
| Q 10 (text; 85) | If available, please name an example of remote monitoring being beneficial/important (e.g. special situation, individual story) | - |
| Q 11 (SC; 504) | Do you get assistance from another person (except your physician) regarding the use of the transmitter | yes/no |
| Q12 (MC/text; 88) | If the answer is yes, who is assisting you? | member of the family/friend/nurse of the cardiological clinic/ambulance/medical employee living in my house/general practitioner/institution/others (text) |
| Q13 A/B (text; 318/388) | How often do you attend in-patient visits to interrogate the CIED  A Before remote-monitoring (months)  B Since remote-monitoring (months) | - |
| Q14 A/B (text; 325/366) | How often do you attend in-patient visits to visit your cardiologist  A Before remote-monitoring (months)  B Since remote-monitoring (months) | - |
| Q15 (text; 205) | How have remote monitoring changed the relationship to your attending cardiologist? | - |
| Q16 (SC; 489) | How did remote monitoring have changed your disease management? | better/worse/constant |
| Q17 (text; 220) | Why? | - |
| Q18 (SC; 494) | Do you experience technical issues regarding your transmitter? | never/very seldomly/sometimes/all the time |
| Q19 (text; 99) | If the answer is yes, please name the individual issues? | - |
| Q20 (SC/text; 459) | Whom do you refer to regarding questions about the transmitter? | cardiologist/relative/association or organization/nobody/manufacturer/emergency service/other medical specialist (text)/other(text) |
| Q21 (SC; 474) | Do you have concerns regarding the remote monitoring? | yes/no |
| Q22 (text; 32) | If the answer is yes, what kind of concerns do you have? | - |
| Q23 (text; 333) | How often do you leave your home for more than 24 hours? | - |
| Q24 (SC/text; 438) | Do you take your transmitter with you if you leave your home for more than 24 hours? | yes, always/yes, if more than 48 hours/yes, if more than one week/no, never/other (text) |
| Q25 (SC; 467) | Do you consider your transmitter as a restriction? | yes/no |
| Q26 (text; 22) | If the answer is yes, can you name examples of those restrictions considered? | - |
| Q27 (SC; 525) | Do you possess a smartphone? | yes/no |
| Q28 (MC/text; 385) | In what kind of situations do you use it? | phone calls/to take pictures/internet/play games/others (text) |
| Q29 (SC; 384) | How often do you use your smartphone? | all the time/few times a day/once a day/few times in a week/(almost) never |
| Q30 (SC) | Do you download applications? | yes/no |
| Q31 (MC/text; 367) | If the answer is yes, what kind of applications do you download? | healthcare (text)/communication and social media/travel/games/media/lifestyle (sports, nutrition…)/ online-shops (shopping)/ items (compass, measurements,…)/office-software (excel, word,…)/banking, insurances/others (text) |
| Q32 (SC; 426) | If you would have the option to use your own smartphone (with an app) to send data regarding your CIED to your attending cardiologist, how big would your interest in such an option be? | 1 (no interest)-10 (big interest) |
| Q33 (text; 219) | What kind of advantages would you ascribe to such an option? | - |
| Q34 (MC/text; 343) | What kind of information should be displayed within such an app? | validation of connectivity/battery status, remaining term of the implantable device/information regarding technical issues/nothing/others (text) |
| Q35 (SC; 447) | Would you prefer a smartphone-based application or a transmitter next to the bed? | smartphone with an app / transmitter / no opinion |
| Q36 (text; 199) | What kind of obstacles would you ascribe to the use of your own smartphone for data transfer of the CIED? | - |
| Q37 (MC/text; 382) | What kind of concerns would you have regarding such an idea? | battery consumption/storage space/data consumption/data safety/power reduction of the smartphone/no concerns/other (text) |

Supplementary table 1: Questionnaire, translated into English language, including the question type (QT), answers to choose as well as the response rate (RR) per question. CIED = cardiac implantable electronic device; SC = Single choice; MC = multiple choice.
